# Network Rewiring: Physiological Consequences of Reciprocally Exchanging the Physical Locations and Growth-Phase-Dependent Expression Patterns of the *Salmonella fis* and *dps* Genes

**DOI:** 10.1128/mBio.02128-20

**Published:** 2020-09-08

**Authors:** Marina M. Bogue, Aalap Mogre, Michael C. Beckett, Nicholas R. Thomson, Charles J. Dorman

**Affiliations:** a Department of Microbiology, Moyne Institute of Preventive Medicine, Trinity College Dublin, Dublin, Ireland; b Wellcome Genome Campus, Hinxton, Saffron Walden, United Kingdom; University of Utah

**Keywords:** nucleoid-associated protein, Fis, Dps, ChIP-seq, RNA-seq, transcriptome, *Salmonella enterica* serovar Typhimurium, virulence

## Abstract

The Fis nucleoid-associated protein controls the expression of a large and diverse regulon of genes in Gram-negative bacteria. Fis production is normally maximal in bacteria during the early exponential phase of batch culture growth, becoming almost undetectable by the onset of stationary phase. We tested the effect on the Fis regulatory network in *Salmonella* of moving the complete *fis* gene from its usual location near the origin of chromosomal replication to the position normally occupied by the *dps* gene in the right macrodomain of the chromosome, and vice versa, creating the gene exchange (GX) strain. In a parallel experiment, we tested the effect of rewiring the Fis regulatory network by placing the *fis* open reading frame under the control of the stationary-phase-activated *dps* promoter at the *dps* genetic location within the right macrodomain, and vice versa, creating the open reading frame exchange (OX) strain. Chromatin immunoprecipitation sequencing (ChIP-seq) was used to measure global Fis protein binding levels and to determine gene expression patterns. Strain GX showed few changes compared with the wild type, although we did detect increased Fis binding at Ter, accompanied by reduced binding at Ori. Strain OX displayed a more pronounced version of this distorted Fis protein-binding pattern together with numerous alterations in the expression of genes in the Fis regulon. OX, but not GX, had a reduced ability to infect cultured mammalian cells. These findings illustrate the inherent robustness of the Fis regulatory network with respect to the effects of rewiring based on gene repositioning alone and emphasize the importance of *fis* expression signals in phenotypic determination.

## Abstract (precis)

We assessed the impact on *Salmonella* physiology of reciprocally translocating the genes encoding the Fis and Dps nucleoid-associated proteins (NAPs) and of inverting their growth-phase production patterns such that Fis was produced in stationary phase (like Dps) and Dps was produced in exponential phase (like Fis). Changes to peak binding of Fis were detected by ChIP-seq on the chromosome, as were widespread impacts on the transcriptome, especially when Fis production mimicked Dps production. Virulence gene expression and the expression of a virulence phenotype were altered. Overall, these radical changes to NAP gene expression were well tolerated, revealing the robust and well-buffered nature of global gene regulation networks in the bacterium.

## INTRODUCTION

*trans*-Acting regulatory proteins play a prominent role in controlling the expression of bacterial genes at the level of transcription. Whether acting positively or negatively, these proteins typically influence their target genes by binding at, or close to, transcriptional promoters (1). Transcription factors differ in the number of genes that are under their control (2), and a majority of these proteins exist with moderate copy numbers of between 1 and 100 monomers (3). Nucleoid-associated proteins (NAPs) play a role in the architecture of the genome, and many contribute to the regulation of transcription (4). NAPs share many of the features of transcription factors, but many are present in thousands of copies. The distinction between “transcription factor” and “NAP” is quite blurry; these are operational descriptions for proteins that lie along a continuum extending from factors with pervasive influences on gene expression to those that have just a few gene targets (5).

Transcription factors must make the journey from the gene that encodes them to the target promoter. This process seems to involve a combination of sliding along DNA and movements between DNA segments in which the interactions with DNA consist of nonspecific and specific binding events (6, 7). In the case of a low-copy-number transcription factor such as the Lac repressor protein, LacI (40 monomers per cell), the addition of an inducer (IPTG [isopropyl-β-d-thiogalactopyranoside]) reduces specific but not nonspecific binding; the protein explores the nucleoid as it searches for its specific binding sites (6). Many NAPs rely on indirect readout to identify their DNA targets, making their binding site selections on the basis of the DNA conformation rather than on the basis of the sequence alone (5). This, combined with their high copy numbers, might allow NAPs to spread rapidly through the nucleoid to locate their DNA targets. Alternatively, their weak requirement for specific base sequences at their DNA targets might increase their dwell time at the many nonspecific sites that they encounter as they migrate through the nucleoid.

The significance of the genomic positions of genes encoding transcription factors or NAPs has been investigated, principally in the model bacterium Escherichia coli. There, a correlation is seen between gene position along the replichore extending from the origin of chromosome replication, *oriC*, to the terminus and the period in the growth cycle when the gene product is most required (8). Gene position also correlates with gene copy number in fast-growing bacteria, when replication recommences before cell division is complete. This correlation is conserved across the *Gammaproteobacteria* (8) suggesting that it is biologically meaningful and may have consequences for the operation of bacterial gene control networks. This hypothesis is supported by previously reported observations suggesting that gene relocations that occur naturally, e.g., through inversions of chromosomal segments, tend to preserve the distance of the affected gene(s) from *oriC* (9–11). It suggests that moving regulatory genes, including genes that encode NAPs, to novel chromosomal locations could result in changes to bacterial physiology. Consistent with this proposal, repositioning the gene encoding the factor for inversion stimulation (FIS) NAP in E. coli alters the cell’s capacity to manage its global DNA topology (12).

Fis is a prominent member of the family of NAPs in Gram-negative bacteria (13, 14) that alters the transcription of hundreds of genes, directly or indirectly and positively or negatively (15–19), and contributes architecturally to site-specific recombination systems (20–22), chromosome replication initiation (23–26), transposon activity (27, 28), and bacteriophage life cycles (22, 27, 29, 30). Fis is not essential, despite its pervasive influence on cell biology, but it enhances the fitness of a wild-type (WT) bacterium when competing with an otherwise isogenic *fis* knockout mutant (31). Among the genes that are regulated by Fis are those encoding components of the translational apparatus of the bacterium, its chemotaxis and motility functions, and many metabolic pathway proteins and (in the case of pathogens) numerous virulence genes (17, 19, 32–34). The association of high Fis concentrations with the early exponential (EE) phase of growth is thought to be indicative of a role for Fis in signaling growth-cycle-related information to the global gene expression program of the cell (31, 35, 36).

The Fis protein influences the topology of DNA both directly, through DNA binding (31), and indirectly, through its influence on the expression of the genes encoding DNA gyrase (*gyrA* and *gyrB*) and DNA topoisomerase I (*topA*) (37–39). The *fis* gene is part of the bicistronic *dusB-fis* operon (40, 41), whose stringently controlled promoter is stimulated by negatively supercoiled DNA, creating a regulatory connection between the global supercoiling level in bacterial DNA, the physiology of the bacterium, and the initiation of *fis* transcription (42). The single *dusB-fis* promoter is autorepressed by the Fis protein (43), with translation of the Fis protein relying on mRNA secondary structure and nucleotide sequence motifs in the upstream message (44).

The production of high levels of Fis protein is associated with the early exponential phase of bacterial growth, with Fis being present at very low levels in the stationary phase (35, 37, 45, 46). This pattern is sensitive to aeration; cultures of Salmonella enterica serovar Typhimurium (*S.* Typhimurium) exhibit sustained, low-level production of Fis into the stationary phase of growth in the absence of aeration (47, 48).

**FIG 1:**
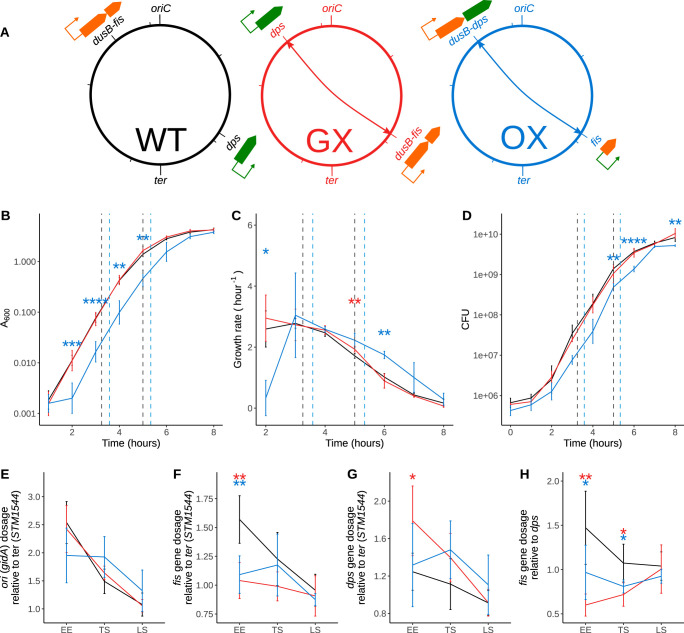
Genomic exchanges of *fis* and *dps* transcription units or ORFs. (A) Schematic depicting wild-type (WT) SL1344 strain with the original locations of *dusB-fis* and *dps* transcription units shown, the constructed gene exchange (GX) strain with the transcriptional units exchanged, and the ORF exchange (OX) strain with only the *fis-dps* ORFs exchanged. (B) Optical density-based growth curves showing the impact of these genetic changes on growth. Early exponential (EE), transition to stationary (TS), and late stationary (LS) time points selected for other analyses are shown. Significance was determined relative to the wild type using Welch's *t* test, and *P* values were adjusted using the Benjamini Hochberg (BH) method. Adjusted *P* values are indicated with asterisks (*, *P* < 0.05; **, *P* < 0.01; ***, *P* < 0.001; ****, *P* < 0.0001). (C) Growth rate curve derived from the OD-based growth curves. (D) CFU-based growth curves. (E) Origin-to-terminus gradients in these strains during the course of the growth curve analysis calculated by assaying the abundance of *gidA* (*oriC* proximal gene) DNA and *STM1544* (*ter* proximal gene) DNA using qPCR. The late stationary (LS) phase is the 24-h time point in the growth curve not shown in the earlier plots. (F) Dosages of the *fis* gene relative to the terminus during the course of the growth curve analysis. (G) Dosages of the *dps* gene relative to the terminus. (H) Dosages of the *fis* gene relative to *dps* analyzed to understand the impact of the gene position swap.

We explored the subtleties of Fis biology by testing the significance of the geographical location of the *fis* gene in the nonstructured left (NS-Left) region of the bacterial chromosome and the physiological significance of the characteristic early-growth-phase-dependent *fis* expression profile. This was achieved by exploiting the chromosome location, promoter, and expression pattern of the *dps* gene. This gene is located in the right macrodomain, where it encodes a ferritin-like, DNA-protecting protein, Dps, which is produced in high concentrations in stationary-phase cells (49–54). A comparison of the proteomes of wild-type E. coli and an isogenic *dps* knockout mutant revealed many differences in protein production, suggesting a role for Dps in the control of protein production at some level (49). Although Dps is a DNA binding protein, it is thought not to influence transcription (51, 55). The *dps* gene is transcribed on entry into stationary phase using RpoS, the stress-and-stationary-phase sigma factor of RNA polymerase, although this can be overridden during oxidative stress when *dps* is transcribed using RpoD in an OxyR- and IHF-dependent mechanism (56, 57). Fis represses *dps* transcription by RNA polymerase containing the RpoD sigma factor, but not RpoS (58). Thus, Fis and Dps production is normally associated with opposite ends of the growth cycle: the early exponential and the stationary phases, respectively (46). The *fis* and *dps* genes are both transcribed in the orientation opposite the direction of DNA replication in the left and right replichores, respectively (Fig. 1A).

We anticipated that altering the expression pattern of the *fis* gene, in a radical manner that fell short of eliminating Fis protein production completely, would provide valuable insights into (i) the robustness or the vulnerability of bacterial physiology in the face of changes to the expression of a prominent NAP gene, (ii) the impact on the operations of the cell of expressing the *fis* gene from a position in the replichore opposite the normal location, and (iii) the significance of the temporal expression pattern of the *fis* gene with respect to the *in vivo* role of the Fis protein. To test our ideas, we relocated the *fis* gene, with its native control elements, from the NS-Left region of the chromosome of *S*. Typhimurium to the *dps* locus in the right macrodomain. In a second genetic exchange, just the protein-encoding, open reading frame (ORF) of *fis* was substituted for that of *dps*, giving *fis* the stationary-phase-specific expression profile of the *dps* gene. Reciprocally exchanging the positions of the entire *dusB-fis* operon and the entire *dps* gene had a relatively modest impact on the physiology of the bacterium. On the other hand, placing *fis* under the control of the *dps* regulatory sequences at the *dps* locus, while driving *dps* gene expression with the *dusB-fis* regulatory sequences at the *dusB-fis* locus, had profound effects on the physiology of the bacterium at the levels of global gene expression, Fis binding patterns around the chromosome, and the ability of the bacterium to infect human cells.

## RESULTS

### Reciprocally exchanging the *fis* and *dps* genes.

The *fis* gene is the downstream component of the bicistronic *dusB-fis* operon (Fig. 1A). We moved the complete *dusB-fis* operon from its usual location near the origin of chromosomal replication to the position normally occupied by the *dps* gene in the right macrodomain of the chromosome, and vice versa, creating the strain GX (gene exchange strain). In a parallel strain construction, we placed the *fis* open reading frame under the control of the stationary-phase-activated *dps* promoter at the *dps* genetic location within the right macrodomain, and vice versa, creating strain OX (open reading frame exchange strain) (Fig. 1A). The construction of the GX and OX strains is described in Materials and Methods (see also Fig. S1 in the supplemental material). The genetic map locations of *fis* and *dps* in the wild-type (WT), GX, and OX strains are summarized in Fig. 1A. We used whole-genome sequencing to rule out the presence of mutations that might have been introduced during strain construction (Fig. S2).

10.1128/mBio.02128-20.1 FIG S1 Schematic representation of the strain construction process. For details, see Materials and Methods. Download FIG S1, EPS file, 0.7 MB. Copyright © 2020 Bogue et al. 2020 Bogue et al. This content is distributed under the terms of the Creative Commons Attribution 4.0 International license.

10.1128/mBio.02128-20.2 FIG S2 Summary of SNP analysis of constructed strains. Heat maps depict summarized Breseq analysis results. (A) Predicted mutations. (B) Unassigned missing coverage evidence. (C) Unassigned new junction evidence. In panels A and B, the color scale indicates the presence (1) or absence (0) of a mutation. In panel C, the color scale indicates the frequency of the new junction among reads mapping to that locus. Download FIG S2, EPS file, 0.9 MB. Copyright © 2020 Bogue et al. 2020 Bogue et al. This content is distributed under the terms of the Creative Commons Attribution 4.0 International license.

The GX and WT strains exhibited similar growth profiles in liquid medium measured using absorbance (Fig. 1B), growth rate (Fig. 1C), or colony forming units (CFU) counts (Fig. 1D). In contrast, the OX strain underperformed in comparison with the WT and GX strains in each of the three growth assessments, with evidence of an extended lag phase being detected (Fig. 1B to D). An exception to this was seen in the case of the growth rate of OX following the transition from exponential growth to stationary phase; here, the growth rate of OX declined more slowly than that of the other two strains (Fig. 1C).

The locations of the *fis* and *dps* genes on the wild-type chromosome are almost diametrically opposite, with *fis* being much closer to the origin of chromosome replication (*oriC*) and *dps* being nearer to the terminus gene, *ter* (Fig. 1A). The gene dosage gradients from *oriC* to *ter* were measured for each of the three strains using quantitative PCR (qPCR) of an origin-proximal gene (*gidA*) and a terminus-proximal gene (STM1544). The measurements were performed at three stages of growth: the early exponential (EE) phase, the transition from exponential growth to stationary phase (TS), and late stationary phase (LS) (for the values of absorbance at 600 nm [*A*_600_], see Materials and Methods). The WT and GX strains had almost identical profiles, while OX had a gradient with a higher variance at each stage of growth than the other two (Fig. 1E). The gene dosage of *fis* was lower in GX and OX than in WT in the EE phase, as expected given its greater proximity to the terminus in the two exchanged strains (Fig. 1F). At the EE phase of growth, the *dps* gene dosage was greater in the GX strain than in the WT, as expected, but was not greater in the OX strain (Fig. 1G), possibly due to the lower growth rate and gene dosage gradient in the OX strain (Fig. 1C) stemming from the lower growth rate of the OX strain (Fig. 1C) at the early time point (Fig. 1E). At the EE phase of growth, the *fis* gene in the GX strain had the lowest gene dosage relative to *dps* (Fig. 1H), thus clearly reflecting the *fis*-*dps* gene position exchange and associated *fis-dps* gene dosage exchange.

### Effects of genetic exchanges on *fis* and *dps* gene expression and protein levels.

**FIG 2:**
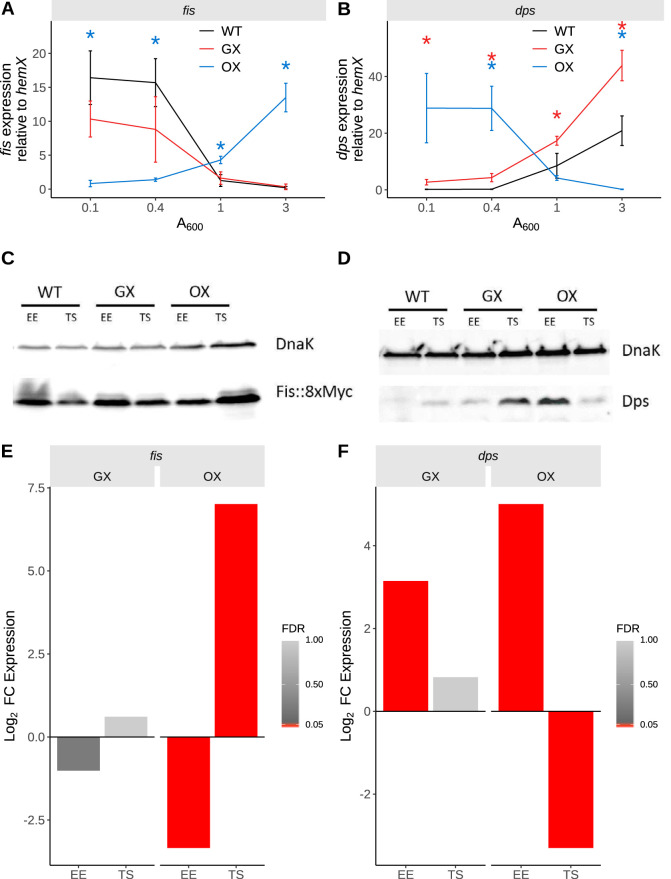
Gene expression changes. (A) Expression of *fis* transcript during the course of the growth curve assayed using RT-qPCR. Significance was determined relative to the wild type using Welch's *t* test, and *P* values were adjusted using the BH method. Adjusted *P* values are indicated with asterisks (*, *P* < 0.05; **, *P* < 0.01; ***, *P* < 0.001; ****, *P* < 0.0001). Black, WT; red, GX; blue, OX. (B) Expression of *dps* transcript during the growth curve. Black, WT; red, GX; blue, OX. (C) Fis protein levels determined by immunoblotting with anti-Fis monoclonal antibody. (D) Dps protein levels determined by immunoblotting with anti-Dps polyclonal serum. (E and F) Expression changes in *fis* and *dps* genes determined by RNA-seq in the GX and OX strains at the EE and TS phases of growth. The color bar indicates FDR, and bars representing genes with FDR values of <0.05 are red.

The transcription of the *fis* (Fig. 2A) and *dps* (Fig. 2B) genes was measured by qPCR using the *hemX* gene as a benchmark. In the WT, each gene was expressed as expected, with *fis* transcription being maximal in early exponential growth (Fig. 2A) and *dps* being transcribed maximally in the stationary phase (Fig. 2B). In the GX strain, both *fis* and *dps* retained their overall expression profiles as seen in the WT but the levels of the *dps* transcript were higher (Fig. 2A and B). In the OX strain, the transcription profile of *fis* was the reverse of that seen in the WT; *fis* transcript levels were highest in stationary phase and lowest in early exponential phase (Fig. 2A). In contrast, *dps* expression was highest in exponential growth and lowest in stationary-phase growth, the reverse of the WT pattern (Fig. 2B). Western blot analyses showed that the patterns of production of Fis (Fig. 2C) and Dps (Fig. 2D) resembled the transcription patterns for the corresponding genes; Dps production in OX mimicked Fis production in the WT, while Fis was produced in OX in a pattern that resembled that of Dps production in the WT. Notably, the expression of *fis* and *dps* genes determined using transcriptome sequencing (RNA-seq) (Fig. 2E and F) matched the expression determined using qPCR (Fig. 2A and B). These data showed that relocating the *dusB-fis* operon or the *dps* gene had a negligible effect on their expression patterns and on the production profiles of their products. However, exchanging just the *fis* and *dps* protein coding regions, with the concomitant switching of cognate regulatory elements, produced reciprocal changes in gene expression and protein production. We next examined the impacts of these changes on Fis protein binding patterns throughout the genome, on the downstream expression of the Fis regulon, and on the physiology of the bacterium.

### Fis binding to the genome in the GX and OX strains.

**FIG 3:**
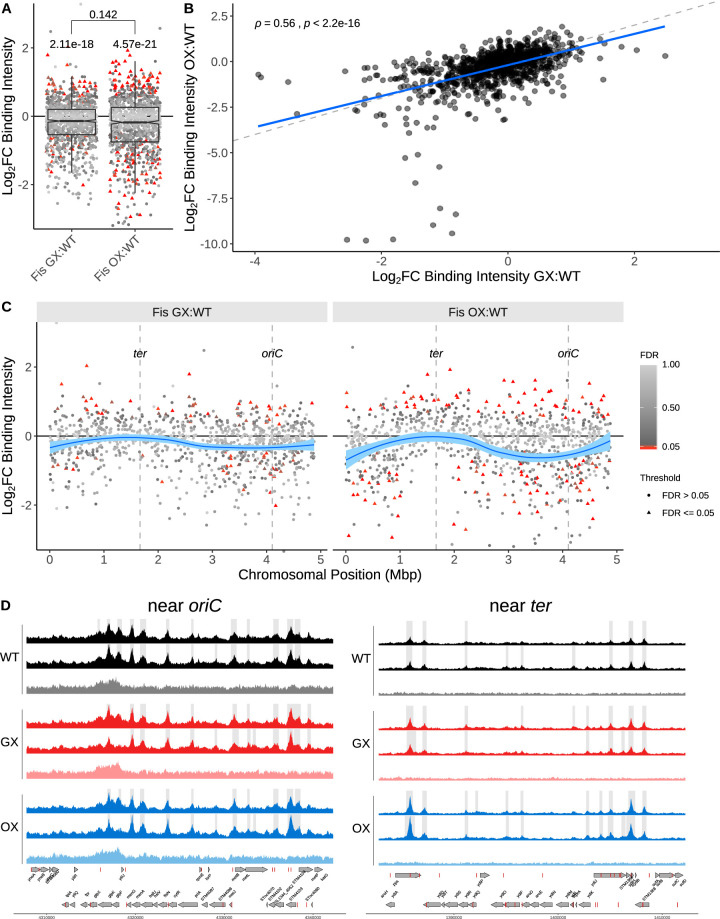
Fis binding changes assayed by ChIP-seq during EE phase. (A) ChIP-seq was used to identify Fis peaks in WT, GX, and OX. The R package DiffBind was then used to determined Log_2_ fold changes in binding intensities of Fis in these peaks in the GX and OX strains relative to the wild type. Significant peak intensity changes (FDR < 0.05) are shown as red filled triangles. To determine if the medians of the distributions of Log_2_ fold changes in binding intensities differed significantly from 0 (no change in Fis binding relative to WT), the Wilcoxon rank sum test was performed with μ = 0; the *P* values are displayed over the individual distributions. The difference between the medians of the GX:WT distribution and the OX:WT distribution was not significant with the Wilcoxon rank sum test as shown above the square bracket corresponding to the data from the single-sample tests (using the less conservative Welch's *t* test here gives a *P* value of 0.0105). (B) Spearman’s correlation of peak intensity changes in the GX in comparison with the OX strain. Dashed line has a slope of 1 and indicates the position where the fold changes in Fis binding intensity in GX would be equal to those in OX. The blue solid line is the regression linear fit to the data. (C) Peak intensity changes along the chromosomal position shown in millions of base pairs. The blue curve shows the local regression peak intensity change calculated using the R locally estimated scatterplot smoothing (LOESS) function. In calculating these intensity changes, the appropriate mock data (WT mock for WT peaks, GX mock for GX peaks, OX mock for OX peaks) were used to control for *ori-ter* gradient changes (Fig. S5). (D) Fis ChIP-seq coverage of the WT, GX, and OX strains (two biological replicates and mock) of example regions near *oriC* and *ter* loci. The ranges of the data indicated on the *y*-axis scale were the same for all the coverage traces and have been omitted for brevity. For each strain, the two biological replicates are represented by the first two coverage traces in the darker color shading. The third, lighter color trace represents the mock coverage for that strain. Gray-shaded regions show peaks called in each sample relative to the mock. Genes (gray arrows) and Fis binding motifs (red blocks) are shown on the positive and negative reference strands. ChIP-seq data for plasmids can be found in Fig. S3.

Chromatin immunoprecipitation sequencing (ChIP-seq) was used to examine the intensity and the distribution of Fis protein binding to the genomes in the GX and OX strains compared to the WT (Fig. 3 [see also Fig. S3]) (Fis motif, Fig. S4). The median Fis binding intensity was lower in both the GX and OX strains than in the WT (Fig. 3A). The intensity of Fis binding changed over a greater range, and was significantly changed at greater numbers of Fis peaks, in the OX strain than in the GX strain (Fig. 3A and C). Changes in Fis binding intensity in the GX strain positively correlated with those in the OX strain, suggesting that these two strains experienced similar changes in Fis binding across genomic loci (Fig. 3B and C). Binding intensity changes showed regional variations along the chromosomes in the OX-GX comparison (Fig. 3C). On average, the reduction in Fis binding was greater around the chromosome origin than around the terminus in both the OX and GX strains. However, the magnitude of the change was greater for OX versus WT than for GX versus WT. In both OX and GX, the Fis protein was being produced from a locus that was closer to the terminus than to the origin (i.e., the gene location was diametrically opposite that in the WT) (Fig. 1A). We also examined the binding patterns of Fis at the negatively autoregulated *dusB-fis* promoter in all three strains. OX and the WT had very similar patterns of Fis binding peaks, but the peaks showed reduced amplitudes in GX (Fig. S6). This may reflect the influence of the new chromosomal neighborhood following the relocation of those Fis binding sites together with *dusB-fis* to a novel chromosomal site (the *dps* locus) in GX.

10.1128/mBio.02128-20.3 FIG S3 Fis binding regions on the chromosome and plasmids. For the SL1344 chromosome, predicted Fis binding regions are shown for the two biological replicates of the WT, GX, and OX strains in early exponential phase. Increased Fis binding around the *ter* gene in the GX and OX strains resulted in peak calling in this region that was slightly better than that seen with the WT. For plasmids, levels of coverage corresponding to the ChIP-seq experiment (solid traces) and predicted Fis binding regions (shaded boxes) are shown for the two biological replicates of the WT, GX, and OX strains in early exponential phase. For comparison, the wild-type mock coverage is also shown as a solid gray trace. Predicted Fis binding motifs are shown in orange, and the motif used for the prediction is briefly mentioned in the legend. This motif is described in detail in the Fig. S4 legend. Chromosomal positions are indicated in millions of base pairs, and plasmid positions are in thousands of base pairs. Download FIG S3, TIF file, 2.9 MB. Copyright © 2020 Bogue et al. 2020 Bogue et al. This content is distributed under the terms of the Creative Commons Attribution 4.0 International license.

10.1128/mBio.02128-20.4 FIG S4 Fis binding motif obtained from ChIP-seq data. A 15-nucleotide-long sequence logo represents the Fis binding motif discovered using MEME on the set of Fis binding regions in the wild-type strain identified using ChIP-seq. Each position in the 15-nucleotide-long palindromic motif is indicated on the *x* axis. The stack of letters at each position represents the most common nucleotides found at that position. The height of each letter represents the relative frequency of that nucleotide at that position in the motif. The overall height of the stack of letters at each position represents the degree of conservation at that position in the motif (if the stack is very short, that position is highly variable). Various statistics reported by MEME are presented below the logo. This motif is similar to the E. coli Fis binding motif. Download FIG S4, EPS file, 0.1 MB. Copyright © 2020 Bogue et al. 2020 Bogue et al. This content is distributed under the terms of the Creative Commons Attribution 4.0 International license.

10.1128/mBio.02128-20.5 FIG S5 Gene dosage gradient differences in the GX and OX strains compared to the WT visualized using coverage data from the ChIP-seq mock experiments performed with these strains. For clarity, the coverage data at each position are not shown; only the local average calculated using the R LOESS function along the chromosomal position in millions of base pairs is shown. There were only slight gene dosage gradient differences in the GX and OX strains at the early exponential time point at which chromatin was collected for ChIP-seq. These differences are considered in the calculation of differentially bound regions. Download FIG S5, EPS file, 0.05 MB. Copyright © 2020 Bogue et al. 2020 Bogue et al. This content is distributed under the terms of the Creative Commons Attribution 4.0 International license.

10.1128/mBio.02128-20.6 FIG S6 Fis binding around *fis* gene. (A) Log_2_ fold changes of Fis binding upstream of the *dusB-fis* transcription unit in GX and OX strains relative to the WT. (B) Fis ChIP-seq coverage of the WT, GX, and OX strains (two biological replicates and mock) of the region around the *fis* gene. The ranges of the *y*-axis scale are the same for all the coverage traces and have been omitted for brevity. For each strain, the two biological replicates are represented by the first two coverage traces in the darker color shade. The third, lighter color trace represents the mock coverage for that strain. Gray-shaded regions show the peaks called in each sample relative to the mock. Genes (gray arrows) and Fis binding motifs (red blocks) are shown on the positive and negative reference strands. The *fis* gene is a part of the *dusB-fis* transcription unit. The promoter of this transcription unit contains two Fis binding motifs, as predicted by FIMO from the MEME suite, and the ChIP-seq data show substantial Fis binding. ChIP-seq data were analyzed using the wild-type reference sequence; thus, the coverage reported here is plotted relative to wild-type gene locations, even though those locations had been altered in the GX and OX strains. In the GX strain, the entire region that included *dusB*, *fis*, and 315 bp of its upstream promoter region was relocated to the *dps* locus. In the OX strain, only the *fis* ORF was relocated to the *dps* locus. In the GX and OX strains, due the strain construction process, an *rrnB* terminator was inserted 63 bp downstream of the *yhdG-fis* operon. This is represented as a dip in the coverage and was expected. Download FIG S6, EPS file, 0.1 MB. Copyright © 2020 Bogue et al. 2020 Bogue et al. This content is distributed under the terms of the Creative Commons Attribution 4.0 International license.

*Salmonella* Typhimurium SL1344 harbors two large plasmids, pSLT (the virulence plasmid) and pCol1B9, both of which are bound by Fis (Fig. S7). As with the chromosome, both plasmids displayed reduced Fis binding at most loci in the GX and OX strains (Fig. S7A) and these changes were positively correlated between the two strains (Fig. S7B).

10.1128/mBio.02128-20.7 FIG S7 Fis binding changes on the *Salmonella* megaplasmids assayed by ChIP-seq during the EE phase. (A) Peak intensity changes along the plasmid position shown in base pairs. In calculating these intensity changes, the appropriate mock data (WT mock for WT peaks, GX mock for GX peaks, OX mock for OX peaks) were used as controls. Significantly differentially bound peaks are roughly annotated with genes that are located in their vicinity. (B) Spearman’s correlation of peak intensity changes in the GX strain in comparison with the OX strain. The dashed line has a slope of 1 and indicates the position where the fold changes in Fis binding intensity in GX would be equal to those in OX. The blue solid line represents the regression linear fit to the data. Download FIG S7, EPS file, 0.4 MB. Copyright © 2020 Bogue et al. 2020 Bogue et al. This content is distributed under the terms of the Creative Commons Attribution 4.0 International license.

### The impact of the genetic exchanges on Fis target gene expression.

**FIG 4:**
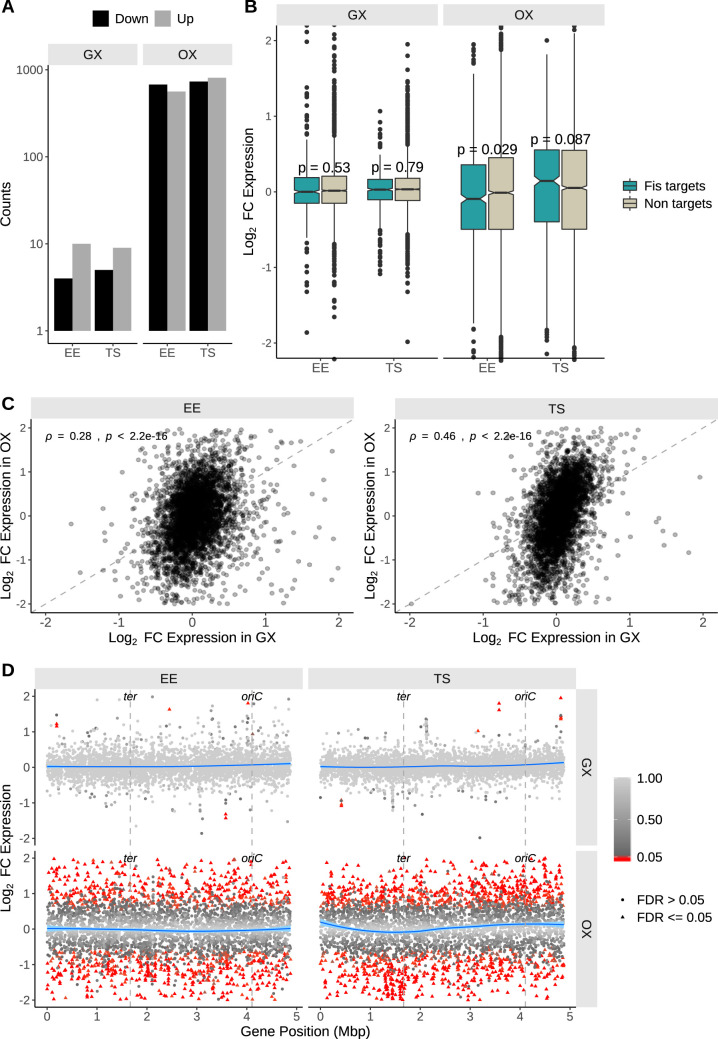
Transcriptomic changes assayed by RNA-seq. (A) Counts of differentially expressed genes. (B) Box plot distribution representations of Log_2_ fold change in expression of genes in the GX and OX strains. The genes have been split into two groups based on whether or not they are predicted to be Fis targets. *P* values obtained using the two-sample Wilcoxon test for the comparison between Fis targets and nontargets are indicated over each pair of box plots. (C) Spearman’s correlation of Log_2_ fold changes in expression of genes in the GX with the OX strain. The dashed line has a slope of 1 and indicates the position where the fold changes are equal. (D) Log_2_ fold change in expression of genes along their position on the chromosome in millions of base pairs. The blue curve shows the local regression peak intensity change calculated using the R LOESS function. Significant gene expression changes (FDR < 0.05) are shown as red filled triangles.

RNA-seq was used to compare the transcriptomes of the GX and the OX strains with that of the WT in cultures at the early exponential phase and at the exponential-phase-to-stationary-phase transition of the growth cycle (Fig. 4). The OX strain showed a higher number of genes with altered expression than the GX strain at both phases of growth (Fig. 4A), and the changes in expression were also greater in the OX strain than in the GX strain (Fig. 4B). Furthermore, the differences between the median values of log_2_ fold change in expression (log_2_FC) of the Fis target genes and the nontarget genes were more significant in the OX strain than in the GX strain. In the OX strain, the median log_2_FC of the Fis target genes was lower in the early exponential phase, and higher in the transition to stationary phase, than that of the nontarget genes. This difference in the levels of Fis target and nontarget gene expression mirrors the greater Fis levels seen during the exponential phase and the lower Fis levels seen in the transition to stationary phase in the OX strain. As with the Fis binding data, we saw slight positive correlations between the GX and OX strains at both the EE and TS phases (Fig. 4C), thus highlighting similarities in the Fis binding changes and associated gene expression changes in the two strains. Plotting the data as a function of position along the chromosome, the OX strain was again found to show a far greater degree of disturbance of the normal expression of its transcriptome than the GX strain compared to the WT, and differentially expressed genes were scattered throughout the chromosome (Fig. 4D). These global gene expression patterns were consistent with the much greater changes seen in *fis* expression, Fis protein production (Fig. 2), and Fis protein binding (Fig. 3) in the OX strain.

**FIG 5:**
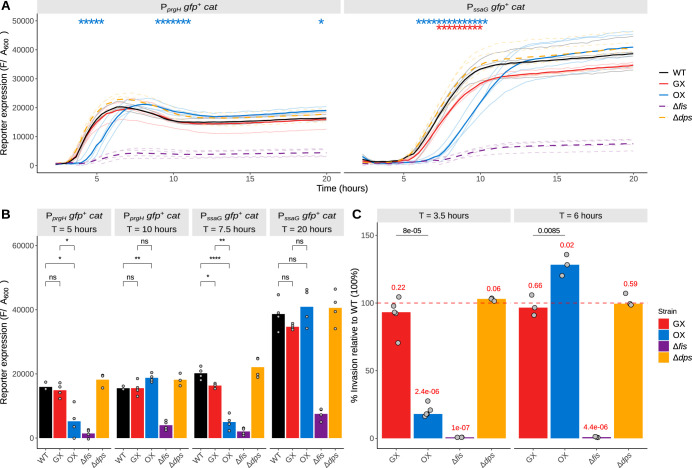
Pathogenicity island expression changes assayed using chromosomal *Pgfp^+^* reporters. (A) SPI-1 expression corresponding to the P*_prgH_ gfp^+^* chromosomal fusions and SPI-2 expression corresponding to the P*_ssaG_ gfp^+^* chromosomal fusions are reported. Reporter expression is measured by determining the fluorescence (F) of *gfp*^+^ expression divided by *A*_600_. Means of results from biological replicates are shown as solid lines, and data from the individual biological replicates are shown in the background as lines with lighter shading. Significance was determined relative to the wild type using Welch's *t* test, and *P* values were adjusted using the BH method. Adjusted *P* values (WT versus GX and WT versus OX comparisons only) are indicated with asterisks (*, *P* < 0.05). (B) A slice of the data in panel A showing reporter expression at early time points (SPI-1, 5 h; SPI-2, 15 h) versus later time points (SPI-1, 15 h; SPI-2, 20 h). Bars represent median values, and the individual biological replicates are represented by the scatter. ns, not significant; *, *P* < 0.05; **, *P* < 0.01; ***, *P* < 0.001; ****, *P* < 0.0001. (C) Results of epithelial cell invasion assay carried out with subcultures grown to two different time points (early, 3.5 h; late, 6 h). Plotted are percent invasions of the strains relative to the WT (100% [not shown], depicted by the red dashed line) in HeLa cells determined 90 min postinfection. Median values of percent invasion are represented by the bars, and values from biological replicates are represented by the scatter. *P* values obtained from one-sample *t* tests (μ = 100) are shown in red above each strain designation. *P* values obtained from Welch’s two-sample *t* tests between GX and OX are indicated over these comparisons in black.

Among the known Fis-dependent genes, those encoding the three type 3 secretion systems found in *Salmonella* exhibited changes in expression (Fig. S8). These genes encode the flagellar apparatus and the *Salmonella* pathogenicity island 1 (SPI-1) and SPI-2 injectisomes. In each case, the largest, most statistically significant effects were seen in the OX strain (Fig. S8). The altered patterns of transcription detected in SPI-1 and SPI-2 in the RNA-seq experiments were investigated in more detail using the following *gfp* reporter gene fusions to representative promoters from each pathogenicity island: P*_prgH_* (SPI-1) and P*_ssaG_* (SPI-2) (Fig. 5A and B). These experiments confirmed that both promoters were dependent on Fis because their full activities were lost in a *fis* knockout mutant. In contrast, the complete removal of Dps from the cell by introducing a *dps* knockout mutation had no effect on either promoter (Fig. 5B). In the OX strain, the SPI-1 P*_prgH_* promoter had reduced activity compared to the WT at 5 h in batch culture but was significantly more active at 10 h (Fig. 5A and B). P*_ssaG_* from SPI-2 was also less active in the OX strain at earlier stages of growth but showed no significant differences from its activity in the WT at 20 h (Fig. 5A and B). The P*_prgH_* promoter was as active in the GX strain as it was in the WT at all stages of growth; the P*_ssaG_* promoter differed from the WT by being less active at the 7.5-h time point.

10.1128/mBio.02128-20.8 FIG S8 Fold changes of some gene sets in the GX and OX strains. (A and B) Motility genes (A) and SPI-1 and SPI-2 genes (B). Expression changes in early exponential (EE) phase and in the transition to the stationary (TS) phase are shown. None of these gene sets were significantly differentially expressed in the GX strain. Note that some of the class 3 motility genes (*fliC, fljA*, and *fljB*) were differentially expressed due to phase switching in the GX and OX strains during their construction. The orientations of these genes in the constructed strains were different from those seen in the wild-type strain against which the gene expression changes were calculated, thus resulting in phase-variation-related gene expression changes. It is unlikely that these changes were related to our study. A general pattern of reversal in gene expression between the early exponential phase and the transition to the stationary phase was seen in the OX strain (this might have been related to Fis levels being higher in the stationary phase in this strain). The color bar indicates FDR, and bars representing genes with FDR values of <0.05 are red. Download FIG S8, EPS file, 0.7 MB. Copyright © 2020 Bogue et al. 2020 Bogue et al. This content is distributed under the terms of the Creative Commons Attribution 4.0 International license.

Given the changes to SPI-1 and SPI-2 gene expression detected in the OX strain, this strain and the GX and WT strains were compared in an *in vitro* infection assay using cultured HeLa cells. The OX strain exhibited a strong reduction in infectivity at the 3.5-h time point, although not as strong as that seen with the *fis* knockout mutant control (Fig. 5C). In contrast, the GX strain showed a modest reduction in invasiveness compared to the WT at both the 3.5-h and the 6-h time points. The OX strain showed enhanced invasiveness at 6 h (Fig. 5C). The *dps* knockout mutant was indistinguishable from the wild type in this respect at either time point.

We also examined the transcriptomes of the two large plasmids in the GX, OX, and WT strains (Fig. S9). As with the chromosome, genes on the pSLT and pCol1B9 plasmids had shown changes in expression in the OX strain, but not in the GX strain, compared with the WT, indicating that the changes made to Fis production in OX had effects beyond the chromosome (Fig. S9). Notably, in the OX strain at the TS phase, we saw an upregulation of the *tra* genes on pCol1B9.

10.1128/mBio.02128-20.9 FIG S9 Transcriptomic changes on the *Salmonella* megaplasmids assayed by RNA-seq. Data represent Log_2_ fold changes in expression of genes along their positions on the plasmid in base pairs. The blue curve shows the local regression peak intensity change calculated using the R LOESS function. Significant gene expression changes (FDR < 0.05) are shown as red filled triangles. Download FIG S9, EPS file, 0.5 MB. Copyright © 2020 Bogue et al. 2020 Bogue et al. This content is distributed under the terms of the Creative Commons Attribution 4.0 International license.

## DISCUSSION

We have investigated the effect of repositioning the *fis* gene on the *S.* Typhimurium chromosome. This gene encodes the conditionally abundant nucleoid-associated protein Fis, which is present in over 50,000 copies in rapidly growing bacteria but in very few copies in stationary-phase cells (35). Previous work investigating the effects of regulatory gene translocation had used *lacI*, the gene encoding the transcriptional repressor, LacI, present in approximately 40 monomers per cell (6, 59). Fis has a much larger regulon than LacI, influencing the transcription of hundreds of genes, positively or negatively (19), and it plays a role in organizing the local structure of the nucleoid (60, 61). Its influence extends beyond transcription and includes effects on chromosome replication (26), transposition (28), and site-specific recombination (20, 22). We sought to investigate if, due to the pervasive influence of Fis, altering the location of the *fis* gene might produce significant changes to bacterial physiology. We were encouraged in our investigation by a previous study in which the translocation of the *dusB-fis* operon to the Ter region of the E. coli chromosome (without repositioning the *dps* gene) correlated with changes to the topology of a reporter plasmid and stress resistance, albeit without alterations to Fis protein levels in the cell (12). The lack of alteration in Fis protein levels was a consequence of an increase in *fis* expression, which compensated for the reduction in the *fis* gene copy number (12). In our study, the same effect was not observed in the GX strain: *fis* expression levels and Fis concentrations remained similar to those of the WT strain in the EE phase, despite the reduced *fis* gene dosage at this point in growth. Furthermore, the GX strain did not exhibit a growth defect whereas the E. coli strain with a translocated *dusB-fis* operon, described previously by Gerganova et al. (12), did. Together, these data indicate that other factors (including bacterial species differences) affect *fis* expression at a given location on the bacterial chromosome. Among these species-specific factors may be the known differences in DNA supercoiling set points that exist between E. coli and *S.* Typhimurium (62–64). The impact of Fis on the homeostatic control of DNA supercoiling, either through its influence on topoisomerase gene expression (36–39) or via direct Fis-mediated buffering of local DNA topology (31), may account for the changes to the transcriptome that we detected. In addition to *dusB-fis* translocation, in our experiments we also studied the effects of rewiring the *fis* gene so that it had the expression profile of *dps*, a gene with a growth-phase expression profile that is diametrically opposite that of *fis* (46, 58). By doing so, we were able to saturate the cell with Fis during stationary-phase growth, a period when Fis is normally undetectable. Similarly, we were able to reduce the production of Fis to a very low level in EE (early exponential) phase, an interval when the protein is normally very abundant.

DNA-binding regulators of transcription and of other molecular processes must communicate with their genomic targets by translocation through the cell, and some studies based on the LacI protein have suggested that the distance to be travelled influences the efficiency of the regulatory connection (65–67). There is some evidence that translated mRNA is spatially constrained in bacteria, remaining close to the site of transcription (68), while other work has suggested that the localization of mRNA is driven by the nature of the protein product and its cellular destination (69, 70). Collectively, these studies suggest that the physical location of a gene in the folded chromosome may determine the influence exerted by its protein product in the cell. A corollary to this proposition is that the repositioning of a gene may alter the sphere of influence of its product.

Our results obtained with the GX strain show that moving the entire *dusB-fis* operon to the right macrodomain site normally occupied by the *dps* gene had relatively modest effects on *Salmonella* physiology. This was true in terms of the growth kinetics of GX compared with the growth kinetics of the wild type (Fig. 1B to D), in the transcriptomic comparison (Fig. 4), and in comparisons of the GX strain and the wild type in terms of SPI-1 *prgH* virulence gene expression (Fig. 5A and B). In contrast, the activity of the SPI-2 virulence gene promoter P*_ssaG_* was significantly reduced in GX compared to the wild type and GX exhibited a reduction in HeLa cell invasion (Fig. 5). Both the P*_prgH_* and the P*_ssaG_* promoters were shown previously to have reduced activity in a *fis* knockout mutant of *Salmonella*, indicating a dependence on this NAP for normal function (19). Our new data show that altering the physical location of the *fis* gene influences P*_ssaG_* negatively and is associated with a reduction in a virulence phenotype. In GX, the *dusB-fis* operon is moved from the nonstructured left region of the chromosome, where SPI-1 is also located, to the right macrodomain, adjacent to the right-hand segment of the Ter macrodomain where SPI-2 is found. The production of the Fis protein in GX increased at the transition to stationary-phase growth compared to the wild type (Fig. 2C), perhaps reflecting the homeostatic resetting of Fis levels reported previously when the *dusB-fis* operon was moved to a Ter-proximal site in E. coli (12). Looking in detail at the SPI-1 and SPI-2 pathogenicity islands, the SPI-2 gene cluster showed the greatest decrease in expression in the OX strain at the onset of stationary phase (see Fig. S8 in the supplemental material), which is consistent with the impact on virulence (Fig. 5C).

Overall, moving the *fis* gene to the native chromosomal location of *dps* and expressing *fis* using the regulatory elements normally used by *dps* in the OX strain had much more profound effects on *Salmonella* physiology than were seen in the GX strain. These effects included an extended lag phase in the OX strain during growth in batch culture (Fig. 1); a much more profound impact on the transcriptome, with many more genes in OX being affected than in GX and with a greater amplitude of positive and negative changes in transcription (Fig. 4); and a greater influence of the expression of key virulence genes and of a virulent phenotype (Fig. 5). The OX strain produced the Fis protein with a profile equivalent to that of Dps in the wild type and expressed Dps in a Fis-like manner (Fig. 2). In E. coli, producing Fis at high levels late in the growth cycle slows growth (71). In contrast, the *Salmonella* OX strain, with its extended lag phase, did not exhibit a growth defect later in the growth cycle (Fig. 1), indicating another point of difference with E. coli. Dps has a negligible impact on transcription (51), so the impact on gene expression in OX is likely to reflect differences in Fis protein levels, a proposition that is supported by the finding that many known Fis targets are among the genes showing altered expression.

Can the changes to *Salmonella* physiology in the GX and OX strains be explained by novel patterns of Fis distribution along the chromosome? The ensemble experiments conducted in this study relied on ChIP-seq to monitor Fis protein distributions during the early exponential phase of growth in living cells. The intensities of the protein binding peaks did change in the two rewired strains, especially in the vicinity of Ter, with OX showing by far the greater change compared to the wild type (Fig. 3C and D). This likely reflects the more radically altered pattern of Fis production in OX, where the transcription signals of the *dps* gene were driving the expression of *fis* (Fig. 1A). Changes to DNA topology caused by altered Fis production may also influence the Fis binding patterns that we detect in our strains (72), but the relative contributions of Fis abundance and target site topology are very difficult to tease apart in global surveys using ensemble experimental methods. The bacterium used in this study, *S.* Typhimurium strain SL1344, also harbors the large plasmids pSLT (a virulence plasmid) and pCol1B9. These autonomously replicating circular DNA molecules are independent of the chromosome, and they also contain Fis binding sites. The patterns of Fis binding to the two plasmids were generally similar in the GX, OX, and wild-type strains (Fig. S3). Plasmid gene expression was unaffected in the GX strain compared to the wild type, but statistically significant differences were detected in the OX strain (Fig. S9).

With the exception of H-NS (73, 74), no NAP in *Salmonella* is essential for the life of the bacterium, and this includes Fis and Dps. The contributions made by NAPs seem to be auxiliary to the functions that they influence. Our findings show that the *Salmonella* genome is well buffered against even quite radical changes to the production of Fis and Dps. The reciprocal inversion of their growth-phase-dependent production profiles, such that Fis assumes the pattern that is characteristic of Dps, and vice versa, does not seriously perturb the life of the cell, although the OX strain does exhibit the many physiological changes that have been documented here. Prominent among these are the effects on *Salmonella* plasmid and virulence gene expression and pathogenesis that we detected. The inversion of the SPI expression pattern in the OX strain relative to the WT (Fig. S8B) and the inversion of the pattern of invasion of this strain relative to the WT (Fig. 5C) demonstrate the role of the timing of Fis expression in controlling the normal timing of expression of these islands. These changes are likely to have their most telling effects when the bacterium is experiencing stress or is trying to compete with the wild type, compromising its ability to compete with the wild-type strain.

## MATERIALS AND METHODS

### Bacterial strains, growth media, and chemicals.

The bacterial strains used in this study and their genotypes and sources (where applicable) are listed in Table 1; all strains were derivatives of Salmonella enterica serovar Typhimurium strain SL1344. Unless otherwise stated, the bacterial strains were cultured in Miller lysogeny broth (LB; 1% NaCl). Kanamycin, carbenicillin, ampicillin, and chloramphenicol were used at concentrations of 50 μg/ml, 100 μg/ml, 100 μg/ml, and 35 μg/ml, respectively.

**TABLE 1:** Strains used in this study

Strain	Genotype	Reference(s) and/or description
SL1344	*rpsL hisG46 manXE95V menCL148L*	85, 101
SL1344 *fis*::*kan*	*fis*::*kan*	Kanamycin resistance cassette inserted 63 bp downstream of *fis* ORF
SL1344 *dps*::*cat*	P*_dps_*::*cat*	Chloramphenicol resistance cassette inserted 149 bp upstream of *dps* ORF
SL1344 2xfG	ΔP*_dps_dps*::P*_fis_dusBfis*	*dps* ORF and upstream/downstream regulatory regions replaced with *dusB-fis* operon and regulatory regions
SL1344 2xdG	ΔP*_fis_dusBfis*::P*_dps_dps*	*dusB* and *fis* ORFs and upstream/downstream regulatory regions replaced with *dps* ORF and regulatory regions
SL1344 2xfO	Δ*dps*::*fis*	*dps* ORF replaced with *fis* ORF
SL1344 2xdO	Δ*fis*::*dps*	*fis* ORF replaced with *dps*
SL1344 Δ*fis*	Δ*fis*::*cat*	19
SL1344 Δ*dps*	Δ*dps*	*dps* ORF deletion
SL1344 GX	ΔP*_dps_dps*::P*_fis_dusBfis* ΔP*_fis_dusBfis*::P*_dps_dps*	Exchange of *dusB-fis* and *dps* and their *cis* regulatory regions
SL1344 OX	Δ*dps*::*fis* Δ*fis*::*dps*	Exchange of *fis* and *dps* ORFs
SL1344 *fis*::*8xmyc*	*fis*::*8xmyc*	Deletion of the *fis* stop codon and insertion of 8 copies of the Myc epitope tag separated by a 21-bp linker
SL1344 GX *fis*::*8xmyc*	GX *fis*::*8xmyc*	Deletion of the *fis* stop codon and insertion of 8 copies of the Myc epitope tag separated by a 21-bp linker
SL1344 OX *fis*::*8xmyc*	OX *fis*::*8xmyc*	Deletion of the *fis* stop codon and insertion of 8 copies of the Myc epitope tag separated by a 21-bp linker
SL1344 P*prgH*	P*prgH*::*gfp*^+^::*cat*	GFP^+^ fused to SPI-1 promoter, P*prgH* (99)
SL1344 GX P*prgH*	P*prgH*::*gfp*^+^::*cat*	GFP^+^ fused to SPI-1 promoter, P*prgH*
SL1344 OX P*prgH*	P*prgH*::*gfp*^+^::*cat*	GFP^+^ fused to SPI-1 promoter, P*prgH*
SL1344 Δ*fis* P*prgH*	P*prgH*::*gfp*^+^::*cat*	GFP^+^ fused to SPI-1 promoter, P*prgH*
SL1344 Δ*dps* P*prgH*	P*prgH*::*gfp*^+^::*cat*	GFP^+^ fused to SPI-1 promoter, P*prgH*
SL1344 P*ssaG*	P*ssaG*::*gfp*^+^::*cat*	GFP^+^ fused to SPI-2 promoter, P*ssaG* (99)
SL1344 GX P*ssaG*	P*ssaG*::*gfp*^+^::*cat*	GFP^+^ fused to SPI-2 promoter, P*ssaG*
SL1344 OX P*ssaG*	P*ssaG*::*gfp*^+^::*cat*	GFP^+^ fused to SPI-2 promoter, P*ssaG*
SL1344 Δ*fis* P*ssaG*	P*ssaG*::*gfp*^+^::*cat*	GFP^+^ fused to SPI-2 promoter, P*ssaG*
SL1344 Δ*dps* P*ssaG*	P*ssaG*::*gfp*^+^::*cat*	GFP^+^ fused to SPI-2 promoter, P*ssaG*
DH5α	*fhuA2 lac*Δ*U169 phoA glnV44* Φ*80*' *lacZ*ΔM15 *gyrA96 recA1 relA1 endA1 thi-1 hsdR17*	102
DH5α pBLUESCRIPT- *dusB-dps-cat*	pBLUESCRIPTSK(+)::*dusB*::*dps*::*cat*	*dusB-dps* operon with *cat* antibiotic resistance gene

### Strain construction.

All strains generated during this study were constructed using phage λ Red recombinase-mediated homologous recombination (75) and P22 transduction (see summary schematic in Fig. S1 in the supplemental material). To construct the gene exchange (GX) and open reading frame exchange (OX) strains, first, the FLP recombination target (FRT)-flanked kanamycin resistance gene (*kan*), amplified from pKD4, was inserted 62 bp downstream of the *fis* ORF (SL1344 *fis*::*kan*) and the FRT-flanked chloramphenicol resistance gene (*cat*), amplified from pKD3, was inserted 190 bp upstream of the *dps* ORF (SL1344 *dps*::*cat*). These strains were used as templates to amplify either *fis*::*kan* ORF or the *dusB-fis*::*kan* and *dps*::*cat* transcriptional units. SLiCE (76) was used to generate a *dusB-dps-cat* construct on the plasmid pBluescript SK(+). This plasmid was used as a template to amplify the *dusB-dps-cat* transcriptional unit (where the *fis* ORF is replaced by the *dps* ORF). As previously described, these amplicons were used to replace the reciprocal gene, generating the 2× intermediate strains in the process that harbored two copies of either the *fis* or *dps* transcriptional units or ORFs (Table 1; see also Fig. S1). P22 phage-mediated generalized transduction was used to generate the GX and OX strains. Finally, the FRT-flanked antibiotic resistance genes were eliminated using a helper plasmid (pCP20) encoding FLP recombinase.

Strain variants of SL1344 and the exchange strains expressing 8× Myc-epitope-tagged Fis were constructed using a modified version of phage λ Red recombinase-mediated recombination (75).

The DNA oligonucleotides used in this study are listed in Table S1 in the supplemental material.

10.1128/mBio.02128-20.10 TABLE S1 List of oligonucleotides used in this study. The nucleotide sequences are written 5′ to 3′. Download Table S1, DOCX file, 0.1 MB. Copyright © 2020 Bogue et al. 2020 Bogue et al. This content is distributed under the terms of the Creative Commons Attribution 4.0 International license.

### Bacterial growth measurement.

Bacterial cell density was measured using absorbance and viable counts.

Absorbance was measured, using a Thermo Scientific BioMate 3S spectrophotometer, at 600 nm (*A*_600_) using sterile LB for blank correction. Cell densities with *A*_600_ values greater than 1 were diluted 1:10 in sterile LB before measurement.

Viable counts were performed by plating serial 10-fold dilutions of the culture made in sterile phosphate-buffered saline (PBS) on sterile LB agar plates. Colonies obtained after overnight incubation at 37°C were counted and multiplied by the appropriate dilution factors to give the viable cell density in terms of CFU.

Overnight cultures were grown for at least 16 h in 4 ml LB in tubes maintained at an angle at 37°C with 200-rpm orbital shaking. For performing growth experiments, overnight cultures were diluted to an *A*_600_ of 0.003 in 25 ml LB in 250-ml conical flasks. These were incubated at 37°C with 200 rpm orbital shaking.

For all experiments, the bacterial cultures were harvested at the early exponential (EE; *A*_600_ of 0.4) phase, transition-to-stationary (TS; *A*_600_ of 3) phase, or late stationary (LS; *A*_600_ of 3.5, 24 h from inoculation) phase of growth.

### Estimating gene dosages using quantitative reverse transcription-PCR (RT-qPCR).

Bacterial chromosomal DNA was isolated by harvesting 1 ml of cultures grown to the EE, TS, and LS phases of growth by centrifugation at 16,000 × *g* for 1 min. The cell pellet was resuspended in sterile distilled water, boiled at 100°C for 5 min, and subjected to vortex mixing, and cell debris was pelleted by centrifugation again. The supernatant was mixed with 1 volume of chloroform, and subjected to vortex mixing. The upper aqueous layer was isolated by centrifugation at 16,000 × *g* for 10 min. The concentration was determined using a DeNovix DS-11 spectrophotometer using *A*_260_ and adjusted to 100 ng/μl with nuclease-free water.

The genomic DNA (gDNA) was probed using primers specific to target genes in a StepOnePlus real-time PCR system. Each 20-μl reaction mixture, set up in the wells of a 96-well plate, contained 1× FastStart Universal SYBR green Master (Rox) (Merck, Wicklow, Ireland), 0.6 μM concentrations of a primer pair, 8 μl gDNA, and sterile distilled water added to reach a volume of 20 μl. For each primer pair, a standard curve of 10-fold serially diluted SL1344 gDNA was included. Threshold cycle (*C_T_*) values of gDNA samples were checked against the standard curves to ensure that they fell in the linear range for each primer pair, and the concentrations of gDNA in the samples were estimated from the standard curve. Cycle conditions for qPCRs were as follows: 95°C for 10 min, followed by 40 cycles of 95°C for 15 s and 60°C for 1 min.

Oligonucleotide primers were designed against an *oriC* (*gidA*) proximal gene and a Ter proximal gene (*STM1554*). To determine the gene dosage gradient, the ratio of *oriC*-proximal DNA to Ter-proximal DNA was identified. Specific gene dosages of *fis* and *dps* were determined by comparing the quantities of these genes to that of *STM1554.*

### Estimating gene expression changes using quantitative reverse transcription-PCR (RT-qPCR).

At optical densities of 0.1, 0.4, 1, and 3, further growth was halted and intracellular RNA was stabilized by the addition of a 0.4 volume of stop solution (5% [vol/vol] phenol [pH 4.3]–ethanol) and incubation on ice for 30 min. A volume of cells equivalent to a cell density of 1 ml of culture at an *A*_600_ of 1 was harvested. Cells were pelleted by centrifugation at 3,220 × *g* for 10 min at 4°C. Cells were resuspended in Tris-EDTA buffer (TE; pH 8.0) containing 50 mg/ml lysozyme. RNA was extracted using an SV total RNA isolation kit (Promega, WI, USA). RNA was DNase treated using a Turbo DNase kit (Invitrogen). RNA integrity was assessed on a HT gel (77) and quantified using a DeNovix DS-11 spectrophotometer and *A*_260_.

RNA (400 ng) was reverse transcribed into cDNA using random oligonucleotides and a GoScript reverse transcriptase kit (Promega). The cDNA was probed using primers specific to target genes in the StepOnePlus real-time PCR system as described above. The *hemX* gene was used as a control gene (with unchanged expression assumed), and the changes in expression of the other genes were calculated against *hemX* expression.

The oligonucleotide primer pairs used in qPCR are listed in Table S1.

### Estimating protein levels using immunoblotting.

At the EE phase or TS phase of growth, a volume of cells equivalent to a cell density of 1 ml of culture with an A_600_ of 1 was harvested and resuspended in 350 μl PBS and transferred to a sonication tube. Cells were lysed by sonication with 10 cycles, with each cycle consisting of 10-s bursts (10-μm amplitude) followed by 30 s of incubation on ice. Samples were diluted with equal volumes of 2× Laemmli sample buffer (final concentrations, 4% SDS, 20% glycerol, 10% 2-mercaptoethanol, 0.004% bromophenol blue, and 0.125 M Tris HCl, pH 6.8) and heated at 100°C for 5 min prior to loading on a 12.5% SDS-PAGE gel.

Proteins were separated by electrophoresis in running buffer (25 mM Tris-HCl, 190 mM glycine, 0.1% [wt/vol] SDS) at 60 V through the stacking gel, which increased to 130 V as the dye moved through the resolving gel until the dye reached the base of the gel, and the proteins were then transferred onto methanol-activated polyvinylidene difluoride (PVDF) membranes (0.2-μm pore size) in transfer buffer (20% [vol/vol] methanol, 25 mM Tris, 190 mM glycine) at 300 mA for 1 h 45 min using a Trans-Blot apparatus (Bio-Rad).

Blocking of the membrane was performed for 1 h at room temperature with a blocking solution (5% skimmed milk powder, phosphate-buffered saline–0.1% Tween 20 [PBST]) and with rocking. Membranes were probed overnight at 4°C using the following primary antibody concentrations: 1:50,000 mouse anti-DnaK monoclonal antibody (Abcam; ab69617), 1:50,000 mouse anti-C-Myc monoclonal antibody (Sigma; M4439), and 1:2,000 rabbit anti-Dps polyclonal serum (Regine Hengge, Humboldt Universitat zu Berlin). All antibodies were diluted to the appropriate concentration in PBST containing 5% bovine serum albumin (BSA). Membranes were washed with PBST for 5 min each while rocking. Horseradish peroxidase (HRP)-conjugated secondary antibodies were diluted 1:10,000 and 1:2,000 for polyclonal goat anti-mouse (Bio-Rad; 170-6516) and goat anti-rabbit HRP (Dako; P0448), respectively. Secondary antibodies were diluted to the appropriate concentration in PBST containing 5% skimmed milk powder (Lab M). Membranes were incubated with secondary antibodies for 1 h 30 min at room temperature while rocking. Membranes were then washed three times with PBST and once with PBS. Blots were incubated in ECL reagent (Pierce) for 1 min, and bands were visualized using an ImageQuant LAS 4000 scanner (GE Healthcare).

### Whole-genome sequencing (WGS) and single nucleotide polymorphism (SNP) analysis to check constructed strains.

**(i) DNA extraction.** Chromosomal DNA for whole-genome sequencing was prepared by a phenol-chloroform method which caused minimal shearing to DNA. Briefly, 2 ml of an overnight culture was harvested by centrifugation at 16,000 × *g* and the cell pellet was resuspended in TE (pH 8.0) (100 mM Tris-HCl [pH 8.0], 10 mM EDTA [pH 8.0]). Lysis was performed by the addition of 1% (wt/vol) SDS and 2 mg/ml proteinase K and incubation at 37°C for 1 h. DNA was isolated by the addition of 1 volume of phenol (pH 8.0):chloroform:isoamyl alcohol (25:24:1), followed by centrifugation at 16,000 × *g* to separate the aqueous and organic layers. Following this, 1 volume of chloroform was added to the aqueous layer, and centrifugation was repeated. To remove contaminants, 0.3 M sodium acetate (pH 5.2) and 5 volumes of 100% (vol/vol) ethanol were added followed by precipitation at −20°C for 1 h. The DNA was pelleted by centrifugation, washed once with 70% (vol/vol) ethanol, dried at 65°C for up to 5 min, and resuspended in 50 μl sterile distilled water. Total gDNA was treated with 100 mg/ml RNase A at 37°C for 30 min. Following this, DNA was isolated by the addition of 1 volume of phenol (pH 8.0):chloroform:isoamyl alcohol as described above.

**(ii) Library preparation.** Library preparation and sequencing for all strains except OX and 2xdO were performed by MicrobesNG (http://www.microbesng.uk), which is supported by the BBSRC (grant number BB/L024209/1). DNA was quantified in triplicate with a Quant-iT dsDNA HS (double-stranded DNA high-sensitivity) assay kit in an Eppendorf AF2200 plate reader. Genomic DNA libraries were prepared using a Nextera XT library prep kit (Illumina, San Diego, USA) following the manufacturer’s protocol with the following modifications: 2 ng of DNA was used as the input instead of 1 ng, and PCR elongation time was increased to 1 min from 30 s. DNA quantification and library preparation were carried out on a Hamilton Microlab Star automated liquid-handling system. Pooled libraries were quantified using a Kapa Biosystems library quantification kit for Illumina on a Roche LightCycler 96 qPCR machine. Libraries were sequenced on an Illumina HiSeq sequencer using a 250-bp paired-end protocol. Reads were subjected to adapter trimming using Trimmomatic 0.30 with a sliding-window quality cutoff of Q15 (78). *De novo* assembly was performed on samples using SPAdes version 3.7 (79), and contigs were annotated using Prokka 1.11 (80).

For the OX and 2xdO strains, library preparation was performed by the Sanger Institute (Hinxton, United Kingdom). Samples were quantified with a Biotium AccuClear ultra-high-sensitivity dsDNA quantitative kit using a Mosquito LV liquid-handling platform, an Agilent Bravo WS automation system, and a BMG FLUOstar Omega plate reader and were subjected to cherry picking to achieve 200 ng/120 μl using a Tecan liquid-handling platform. Cherry-picked plates were sheared to 450 bp using a Covaris LE220 instrument. After shearing, the samples were purified using Agencourt AMPure XP solid-phase reversible immobilization (SPRI) beads on an Agilent Bravo WS platform. Library construction (end repair [ER], A-tailing, and ligation) was performed using a NEB Ultra II custom kit on an Agilent Bravo WS automation system. The PCR system was set up using Kapa HiFi Hot Start mix and IDT 96 iPCR tag barcodes on Agilent Bravo WS automation system. The PCR cycles (6 standard cycles) were performed as follows: (i) 95°C for 5 min, (ii) 98°C for 30 s, (iii) 65°C for 30 s, (iv) 72°C for 1 min, (v) five repeats of cycles ii to iv, and (vi) incubation at 72°C for 10 min, followed by post-PCR plate purification using Agencourt AMPure XP SPRI beads on a Beckman BioMek NX96 liquid-handling platform. Libraries were quantified with a Biotium AccuClear ultra-high-sensitivity dsDNA quantitative kit using a Mosquito LV liquid-handling platform, a Bravo WS platform, and a BMG FLUOstar Omega plate reader. Libraries were pooled in equimolar amounts on a Beckman BioMek NX-8 liquid-handling platform. Libraries were normalized to 2.8 nM such that they were ready for cluster generation on a c-Bot cluster generation system and loading on an Illumina sequencing platform.

**(iii) SNP analysis.** SNP analysis was performed using Breseq (81–83). This is a pipeline that performs all necessary functions, including mapping reads using Bowtie2 (84) and calling SNPs and other genomic variations.

The SL1344 parental strain used in this study has two previously described SNPs, in *manX* (E95V) and *menC* (L148L), compared to the reference genome (85) (Fig. S2). While no additional SNPs were identified in the WT strain or the GX strain, the OX strain contained additional SNPs in *ybiH* (V173L) and *SL1344_3357* (C260C) and in the intergenic region between *SL1344_3765* and *emrD* (Fig. S2). None of the genes with SNPs were found to interact with either Fis or Dps by Search Tool for the Retrieval of Interacting Genes/Proteins (STRING) analysis (86).

### Revealing transcriptomic changes with RNA-seq.

**(i) RNA extraction.** At the EE phase or TS phase of growth, cells were harvested as described for RT-qPCR. The bacterial pellet was dissolved in TE buffer (pH 8.0) (100 mM Tris-HCl [pH 8.0], 10 mM EDTA [pH 8.0]) containing 0.5 mg/ml lysozyme. Lysis was carried out with 1% SDS and 0.1 mg/ml proteinase K during incubation at 40°C for 20 min. RNA was isolated by the addition of 0.3 M sodium acetate (pH 6.5) and 1 volume of phenol (pH 4.3):chloroform (1:1), followed by centrifugation (16,000 × *g*, 4°C, 10 min) in a phase lock tube (Quantabio, VWR) to separate the aqueous and organic phases. Following this, 1 volume of chloroform was added to the aqueous layer and centrifugation (16,000 × *g*, 4°C, 10 min) was repeated in the same phase lock tube. RNA was precipitated by the addition of 5 volumes of 100% ethanol and incubation at −20°C for 1 h. RNA was pelleted by centrifugation (16,000 × *g*, 4°C, 10 min), washed once with 70% ethanol, and resuspended in 50 μl diethyl pyrocarbonate (DEPC)-treated water. RNA was diluted to 500 ng/μl, denatured at 65°C for 5 min, and treated with 10 U RNase-free DNase I (Thermo Fisher Scientific, Waltham, USA) in DNase 1 buffer at 37°C for 40 min. DNase-treated RNA was cleaned up using the phenol chloroform method again. RNA integrity was assessed as described in the paragraph under the RT-qPCR heading.

**(ii) Library preparation.** Strand-specific library preparation and sequencing of the DNase-treated RNA was performed by Vertis Biotechnologie AG (Freising-Weihenstefan, Germany). Briefly, samples were analyzed by capillary electrophoresis (Bioanalyzer, Agilent), and rRNA was depleted using a bacterial Ribo-Zero rRNA removal kit (Illumina). Ribodepleted RNA samples were fragmented using ultrasound (four 30-s pulses at 4°C). Oligonucleotide sequencing adapters were ligated to the 3′ end of each specific strand of the RNA molecules. First-strand cDNA synthesis was performed using Moloney murine leukemia virus (M-MLV) reverse transcriptase and the 3′ adapter as the primer. The first-strand cDNA was purified, and the 5′ Illumina TruSeq sequencing adapter was ligated to the 3′ end of the antisense cDNA in a strand-specific manner. The resulting cDNA was PCR amplified to about 10 to 20 ng/μl using a high-fidelity DNA polymerase. The cDNA was purified using an Agencourt AMPure XP kit (Beckman Coulter Genomics) and was analyzed by capillary electrophoresis (Bioanalyzer; Agilent). For Illumina NextSeq sequencing, the samples were pooled in approximately equimolar amounts. The cDNA pool was subjected to size fractionation in the size range of 200 to 550 bp using a differential cleanup method with the Agencourt AMPure kit. An aliquot of the size-fractionated pool was analyzed by capillary electrophoresis. Samples were PCR amplified for Truseq according to the instructions of Illumina. For the WT and GX strains, the cDNA pool was subjected to paired-end sequencing on an Illumina NextSeq 500 system using 2-by-75-bp read lengths. For the OX strain, the cDNA pool was subjected to single-end sequencing on an Illumina NextSeq 500 system using 1-by-75-bp read lengths.

**(iii) Sequencing alignment.** Raw read sequence qualities were assessed using FastQC (http://www.bioinformatics.babraham.ac.uk/projects/fastqc/). BWA (87) was used to align reads to the Salmonella enterica subsp. *enterica* serovar Typhimurium strain SL1344 chromosome and plasmid reference sequences (chromosome, NC_016810.1; pCol1B9_SL1344, NC_017718.1; pRSF1010_SL1344, NC_017719.1, pSLT_SL1344, NC_017720.1). Aligned reads were sorted by the reference sequence coordinate, and low-mapping-quality reads (mapping quality < 30) were removed using SAMtools (88). SAMtools was also used to summarize mapping statistics. More than 90% of the reads were mapped, and >98% of the mapped reads were unique. Sequence coverages were calculated using Deeptools (89) and are available as BigWig (.bw) files that can be viewed using IGB (90).

**(iv) Differential expression analysis.** Counts of the number of reads mapping to genomic features (genes or small RNA [sRNA]) were obtained using the R package Rsubread (91). The R package EdgeR (92, 93) was used to identify differentially expressed genes. *P* values were adjusted using the Benjamini Hochberg method (false-discovery rate [FDR]), and a cutoff of 0.05 was used to call differentially expressed genes. These are available in an Excel file on GEO.

### Revealing Fis binding changes with ChIP-seq.

**(i) ChIP (chromatin immunoprecipitation).** For all ChIP experiments, *fis*::*8xmyc* epitope-tagged variants of the WT, GX, and OX strains were used (72). Overnight cultures were diluted to a starting *A*_600_ of 0.003 in 25 ml LB broth and grown to an *A*_600_ of 0.4. Cells were harvested by centrifugation at 3,220 × *g* for 10 min at room temperature and resuspended in 50 ml PBS. Formaldehyde was added dropwise to reach a final concentration of 1% (vol/vol) while the cells were stirred continuously. After 10 min of cross-linking, the reaction was quenched with cold glycine (2 M) added dropwise to reach a final concentration of 0.125 M. Cells were stirred further for 5 min and then harvested by centrifugation at 3,220 × *g* for 10 min at 4°C. The pellet was suspended in 600 μl lysis buffer (50 mM Tris-HCl [pH 8.1], 10 mM EDTA [pH 8.0], 1% [wt/vol] SDS, Roche protease inhibitor cocktail) and incubated for 10 min on ice prior to the addition of 1.2 ml IP dilution buffer (20 mM Tris-HCl [pH 8.1], 150 mM NaCl, 2 mM EDTA [pH 8.0], 1% [vol/vol] Triton X-100, 0.01% [wt/vol] SDS, Roche protease inhibitor cocktail) and transferred to a sonication tube. Chromatin was sheared to an average length of 500 bp by sonication at an amplitude of 10 μm 12 times for 30 s each time, with 1 min of incubation on ice between bursts, using a Soniprep 150 instrument. Sheared chromatin was analyzed by agarose gel electrophoresis and stored at −80°C. To preclear chromatin for input samples, 50 μl normal rabbit IgG (Millipore, Cork, Ireland) was added and samples were incubated for 1 h on a model SB1 blood tube rotator at 4°C. Antibody was removed by the addition of 100 μl homogeneous protein G agarose (Roche) and further incubation for 3 h. The protein G agarose was removed by centrifugation at 3,220 × *g* for 2 min, and 200 μl was used as the input. For all ChIP experimentsm a mock-IP system and an experimental IP system were set up at the same time using 1,350 μl precleared chromatin and 10 μg of normal mouse IgG (Millipore) and monoclonal mouse anti-c-Myc, respectively. Samples were incubated at 4°C for 16 h on a model SB1 blood tube rotator. A 100-μl volume of homogeneous protein G agarose was added to each sample, and the samples were incubated for a further 3 h. Protein G agarose beads were pelleted by centrifugation at 5,200 × *g* and washed 4 times with high-salt IP wash buffer (50 mM HEPES [pH 7.9], 500 mM NaCl, 1 mM EDTA, 0.1% [wt/vol] SDS, 1% [vol/vol] Triton X-100, 0.1% [wt/vol] deoxycholate) and twice with TE (pH 8.0). Protein-DNA complexes were eluted from the beads twice with IP elution buffer (100 mM NaHCO_3_, 1% [wt/vol] SDS).

DNA was purified and recovered by performing a standard phenol‐chloroform extraction, followed by ethanol precipitation with 5 μg of glycogen (Invitrogen; catalog no. 10814‐010). The DNA pellets of the IP samples were resuspended (by heating at 37°C) in 50 μl of sterile filtered water for experimental and mock IPs and in 100 μl for the input DNA samples.

**(ii) Library preparation.** ChIP sequencing was performed by Vertis Biotechnologie AG using Illumina NextSeq 500 technology. The DNA samples were first fragmented with ultrasound (2 pulses of 30 s at 4°C). After end repair was performed, TruSeq sequencing adapters were ligated to the DNA fragments. Finally, the DNA was PCR amplified to about 10 to 20 ng/μl using a high-fidelity DNA polymerase. Aliquots of the PCR-amplified libraries were examined by capillary electrophoresis (Bioanalyzer; Agilent). For Illumina NextSeq sequencing, the samples were pooled in approximately equimolar amounts. The DNA pool was eluted in the size range of 250 to 550 bp from a preparative agarose gel. An aliquot of the size-fractionated library pool was analyzed by capillary electrophoresis (Bioanalyzer; Agilent). The adapters were designed for TruSeq sequencing according to the instructions of Illumina. The shotgun library pool was sequenced on an Illumina NextSeq 500 system using 75-bp read length.

**(iii) Sequencing alignment.** See RNA-seq analysis above.

**(iv) Peak calling.** Since we were performing differential binding analysis, we did not exclude duplicate reads as they contain information regarding the binding intensity of the transcription factor being pulled down. MACS2 (94) was used to call peaks against the mock treatment. Each of the strains (WT, GX, and OX) had its own mock sample. We used a custom R script to roughly annotate peaks with the names of neighboring genes and sRNA. These lists are available as both Excel and .gff files. The sequences of Fis peaks from the WT were used to search for motifs using unbiased MEME (95). This Fis motif was then used to search for all possible motifs in the genome, bound or not in the ChIP, using FIMO software (96). This list is also available as a. gff file.

**(v) Differential binding analysis.** Differential binding analysis was performed in R (97) using the DiffBind package (98). To correct for differences in gene dosage gradient between strains, mock treatment samples derived from each strain were used as a control. The output of the differential binding analysis is available (both with and without applying the FDR cutoff) as Excel files.

### *Salmonella* pathogenicity island expression analyses using P*gfp*^+^ reporter fusions.

SPI-1 (P*_prgH_-gfp*^+^) and SPI-2 (P*_ssaG_-gfp*^+^) promoter fusions were integrated into the SL1344 chromosome by transduction by bacteriophage P22 and were selected for on LB agar plates supplemented with 25 μg/ml chloramphenicol (99). Overnight cultures of SPI-1 and SPI-2 reporter fusion strains were diluted 1:100 in LB. These were transferred to the wells of a black 96-well plate with a flat and transparent bottom (Corning, Fisher Scientific) in 4 to 6 technical replicates. The plate was incubated at 37°C for 24 h with 300-rpm (5-mm) shaking in a Synergy H1 microplate reader (Biotek, VT, USA). *A*_600_ and fluorescence (excitation, 485-nm wavelength; emission, 528-nm wavelength) were measured every 20 min. Data were obtained from multiple biological replicates.

### Epithelial cell invasion assay.

The gentamicin protection assay was used as described previously (100) to determine *Salmonella* invasion. HeLa cells (ATCC) were maintained by passaging in complete growth medium (CGM; minimum essential Eagle medium supplemented with 10% [vol/vol] fetal bovine serum, 2 mM l-glutamine, 1 mM sodium pyruvate) every 3 to 4 days (37°C, 5% CO_2_). HeLa cells were seeded 20 to 24 h prior to invasion assay in the wells of a 24-well tissue culture plate at a density of ∼5 × 10^4^ cells in 1 ml CGM.

Overnight cultures (16 h of growth at 37°C, 200 rpm) of the *Salmonella* WT, GX, OX, Δ*fis*, and Δ*dps* strains were diluted 1:33 in 10 ml LB in a 125-ml flask and grown for 3.5 h (early time point) or 6 h (late time point) at 37°C and 200 rpm. Cells were pelleted by centrifugation at 8,000 × *g* for 2 min. A 900-μl volume of the supernatant was discarded without disturbing the pellet and then resuspended in 900 μl Hanks’ buffered saline solution (HBSS^−/−^). Cells were diluted 1:10 in HBSS^−/−^ and then diluted in CGM to form the infection mixture that was used to infect the HeLa cells at a multiplicity of infection (MOI) of 50 with two replicates per strain. No more than three strains were tested at a time, with the WT always being tested as the reference strain. The infection was allowed to proceed for 10 min at 37°C and 5% CO_2_, following which the infection mixture was discarded by rapid aspiration. Cells were washed rapidly with HBSS supplemented with calcium chloride and magnesium chloride (HBSS^+/+^) twice to get rid of extracellular bacteria and were covered with 1 ml CGM. These were incubated at 37°C and 5% CO_2_ for 20 min and then again washed rapidly with HBSS^+/+^ twice and covered with 1 ml CGM supplemented with 50 μg/ml gentamicin to kill any remaining extracellular bacteria. After 1 h of incubation at 37°C and 5% CO_2_, cells were washed rapidly with phosphate-buffered saline (PBS) twice and lysed with 1 ml 2% (wt/vol) sodium deoxycholate–PBS.

Viable counts of the subculture, infection mixture, and lysed cells were obtained. Percent invasion was obtained by calculating the percentage of bacteria in the infection mixture that could invade the cells relative to the WT (counts of intracellular bacteria were calculated relative to the counts of the infection mix to control for variations in infection load and then relative to the wild type to control for day-to-day variation). The one-sample *t* test was used to assess significance relative to the wild type (μ = 100%, 100% being the percentage of invasion of the WT), and the two-sample Welch *t* test was used to assess the significance of pairwise comparisons with α = 0.05. The *P* values from the two-sample tests were corrected for multiple testing using the BH method (FDR).

### Data availability.

RNA-seq and ChIP-seq data can be found on the Gene Expression Omnibus (accession number GSE152228). WGS data can be found on the Sequence Read Archive (accession number PRJNA638833). WGS data for strains OX and 2xdO can be found on the European Nucleotide Archive (accession numbers ERS2515905 and ERS4653304, respectively). Scripts used in the analysis can be found on GitLab (project identifier 10206830 [https://gitlab.com/aalap.mogre/chip-seq-and-rna-seq-in-salmonella-swap-strains]).
