# Supplementary figures and images for: Network Rewiring: Physiological Consequences of Reciprocally Exchanging the Physical Locations and Growth-Phase-Dependent Expression Patterns of the Salmonella fis and dps Genes
Source: mBio. 2020 Sep 8;11(5):e02128-20. doi: 10.1128/mBio.02128-20 (PMC7482072; doi:10.1128/mBio.02128-20)

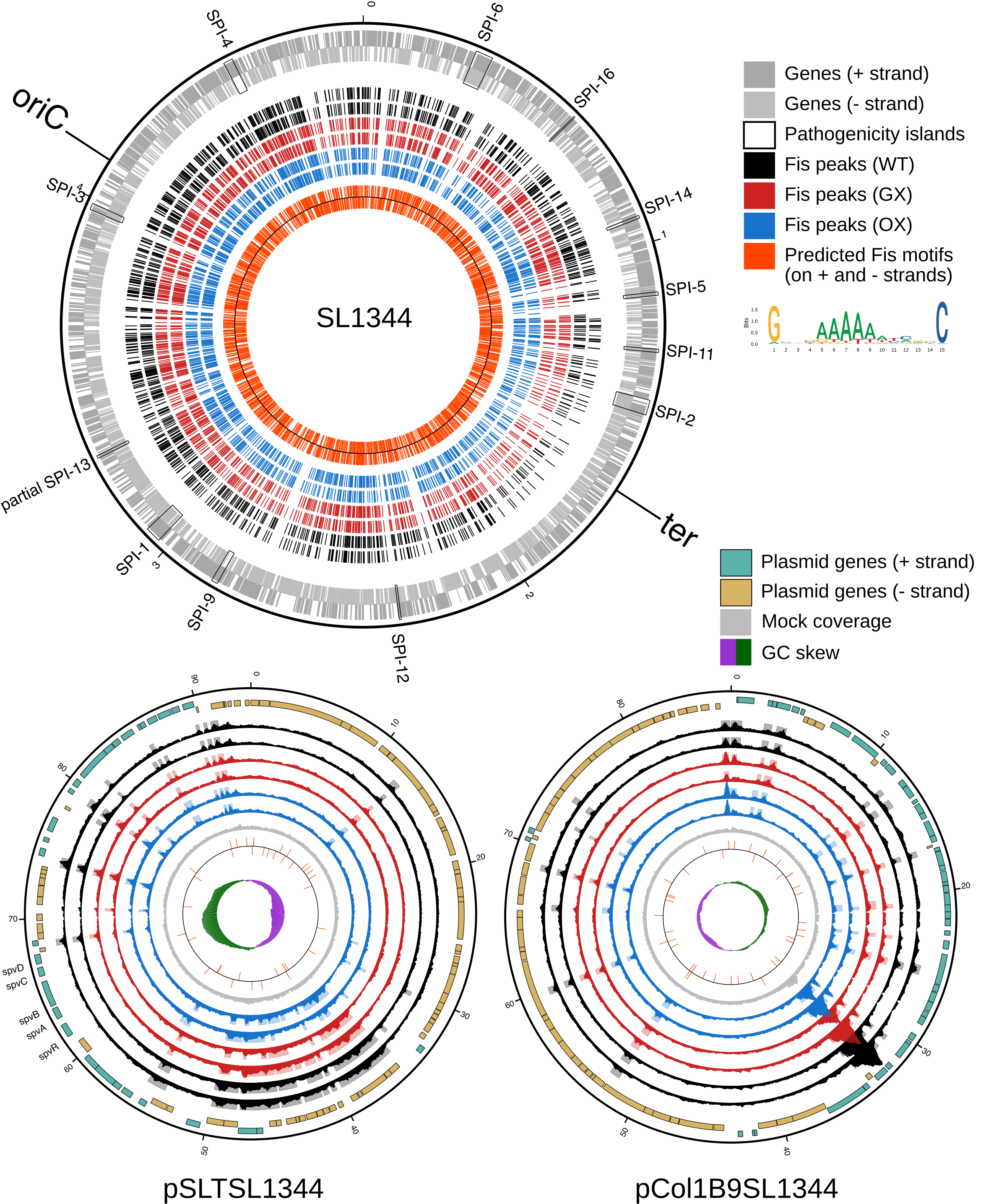

Supplement: FIG S3 [file mBio.02128-20-sf003.tif]
