# Supplementary material for: Network Rewiring: Physiological Consequences of Reciprocally Exchanging the Physical Locations and Growth-Phase-Dependent Expression Patterns of the Salmonella fis and dps Genes
Source: mBio. 2020 Sep 8;11(5):e02128-20. doi: 10.1128/mBio.02128-20 (PMC7482072; doi:10.1128/mBio.02128-20)
Supplement: TABLE S1 [file mBio.02128-20-st001.docx]

**Table S1 Oligonucleotides used in this study.**

| **Name** | **Sequence 5'-3'** |
| --- | --- |
| fisVal-F | GCTGTCCGGGTTGTTCTG |
| fisVal-R | ACCAAATTCCATGTGATGCGT |
| dpsVal-F | CAGTATGCCGCACCGTTT |
| dpsVal-R | GCGCTATTACTTCGTCATTTTTTGT |
| fiskanInsert-F | TAAAAAGGCGCTACTCGGCATGGGGAAGCGCCTTTTTTATGTGCCACCTGCATCGATGGC |
| fiskanInsert-R | TTCACATTCCGCTTTCATGACCAAATTCCATGTGATGCGTCATATGAATATCCTCCTTAG |
| dpscmInsert-F | GACGAGTTTGCTGTTTGGTGGGTAATAATTCTCATTTTAAGTGTAGGCTGGAGCTGCTTC |
| dpscmInsert-R | TACTTCGTCATTTTTTGTCATATTTTTTCTCATTTTTTACCATATGAATATCCTCCTTA |
| fisReg-F | GTCATTTTTTGTCATATTTTTTCTCATTTTTTACATTAAAGTGATCTTGTCCTGAAAC |
| fisReg-R | CAAATAATTCACTTTTGTCGGAGGGGAGTACAAGACGTGTCATATGAATATCCTCCTTAG |
| fisORF-F | TTAATTACCTGGGACACAAACATCAAGAGGATATGAGATTATGTTCGAACAACGCGTA |
| dpsReg-F | TTCACATTCCGCTTTCATGACCAAATTCCATGTGATGCGTCAAGACGTGTGCACTATT |
| dpsReg-R | CTTCCGACTGGCAATGGAGAAAAATCACGCGCAGCGGGAACATATGAATATCCTCCTTAG |
| pBLUE_BB_F | CCAGCTTTTGTTCCCTTTAG |
| pBLUE_BB_R | CCCAATTCGCCCTATAGTG |
| pBLUE_dusB_F | GTGAATTGTAATACGACTCACTATAGGGCGAATTGGGCGATTCATTGATCTACAACA |
| pBLUE_dusB_R | GATTAGACGCTTTTGTTTTTACCAGTTTAGCGGTACTCATAGTTCTGTCAGCTCTTTATTTC |
| pBLUE_dps_F | GAAAATTTTGCGTAAACAGAAATAAAGAGCTGACAGAACTATGAGTACCGCTAAACTGGT |
| pBLUE_dps_R | TAGGAACTTCGGAATAGGAACTAAGGAGGATATTCATATGCAAGACGTGTGCACTATTTA |
| pBLUE_cat_F | TGGCACAGGGGTTTTGCACTTAAATAGTGCACACGTCTTGCATATGAATATCCTCCTTAG |
| pBLUE_cat_R | AAGCTCGAAATTAACCCTCACTAAAGGGAACAAAAGCTGGGTGCCACCTGCATCGATGGC |
| fis8mycins_F | GGTACGCTGCGTAAAAAATTAAAAAAATACGGCATGAACGTCGGATCCAGTCTTCGTGAT |
| fis8mycins_R | GAGTAGCGCCTTTTTAAACAAGCAGTTAGCTAATCGAAAAATTCCGGGGATCCGTCGACC |
| pBLUE_chq_F | GACGTTGTAAAACGACGGCC |
| pBLUE_chq_R | CGGCTCGTATGTTGTGTGG |
| SLiCE_dpsORF_F | ATTTTAATGAATTATAAATTTATTTTGCTGTTTTTAAGCACGATTCATTGATCTACAACA |
| SLiCE_dpsORF_R | TTCACATTCCGCTTTCATGACCAAATTCCATGTGATGCGTGTGCCACCTGCATCGATGGC |
| **qPCR primers** |  |
| RT_fis_F | TGACGTACTGACCGTTTCTACCGT |
| RT_fis_R | ACGTCCTGACCATTCAGTTGAGCA |
| RT_dps_F | CACTGACCGATCATCTGGATAC |
| RT_dps_R | CGGATAGCTTTTCAGTGGAGTT |
| RT_hemX_F | CGCCTGACGGTATGTTTCTT |
| RT_hemX_R | CCCAACCAGGACGTCTATTTAC |
| RT_gidA_F | CAGATTCGGCTGATTCTCCA |
| RT_gidA_R | TCAGGCAGGTATTCAGTTTAGG |
| RT_STM1554_F | TTTAGACGCCCGCACTTC |
| RT_STM1554_R | TAGCTCTCCCGAGTTAGATAATCA |
